# Effects of continuous subcutaneous insulin infusion on the microstructures, mechanical properties and bone mineral compositions of lumbar spines in type 2 diabetic rats

**DOI:** 10.1186/s12891-022-05452-0

**Published:** 2022-05-30

**Authors:** Xiaorong Hu, He Gong, Aiqi Hou, Xiaodan Wu, Peipei Shi, Yingying Zhang

**Affiliations:** 1 Key Laboratory of Biomechanics and Mechanobiology (Beihang University), Ministry of Education, Beijing Advanced Innovation Center for Biomedical Engineering, School of Biological Science and Medical Engineering, Beihang University, Beijing, 100083 China; 2 Beijing Key Laboratory of Rehabilitation Technical Aids for Old-Age Disability, Key Laboratory of Human Motion Analysis and Rehabilitation Technology of the Ministry of Civil Affairs, National Research Center for Rehabilitation Technical Aids, Beijing, 100176 China

**Keywords:** Continuous subcutaneous insulin infusion, T2D, Mechanical properties, Microstructure, Bone mineral composition

## Abstract

**Background:**

Continuous subcutaneous insulin infusion (CSII) for the treatment of type 2 diabetes (T2D) can improve the structure and strength of femur of rats, but the effect of CSII treatment on the lumbar spine of T2D rats is unknown. The purpose of this study is to investigate the effects of CSII on the microstructure, multi-scale mechanical properties and bone mineral composition of the lumbar spine in T2D rats.

**Methods:**

Seventy 6-week-old male Sprague–Dawley (SD) rats were divided into two batches, each including Control, T2D, CSII and Placebo groups, and the duration of insulin treatment was 4-week and 8-week, respectively. At the end of the experiment, the rats were sacrificed to take their lumbar spine. Microstructure, bone mineral composition and nanoscopic-mesoscopic-apparentand-macroscopic mechanical properties were evaluated through micro-computed tomography (micro-CT), Raman spectroscopy, nanoindentation test, nonlinear finite element analysis and compression test.

**Results:**

It was found that 4 weeks later, T2D significantly decreased trabecular thickness (Tb.Th), nanoscopic-apparent and partial mesoscopic mechanical parameters of lumbar spine (*P* < 0.05), and significantly increased bone mineral composition parameters of cortical bone (*P* < 0.05). It was shown that CSII significantly improved nanoscopic-apparent mechanical parameters (*P* < 0.05). In addition, 8 weeks later, T2D significantly decreased bone mineral density (BMD), bone volume fraction (BV/TV) and macroscopic mechanical parameters (*P* < 0.05), and significantly increased bone mineral composition parameters of cancellous bone (*P* < 0.05). CSII treatment significantly improved partial mesoscopic-macroscopic mechanical parameters and some cortical bone mineral composition parameters (*P* < 0.05).

**Conclusions:**

CSII treatment can significantly improve the nanoscopic-mesoscopic-apparent-macroscopic mechanical properties of the lumbar spine in T2D rats, as well as the bone structure and bone mineral composition of the lumbar vertebrae, but it will take longer treatment time to restore the normal level. In addition, T2D and CSII treatment affected bone mineral composition of cortical bone earlier than cancellous bone of lumbar spine in rat. Our study can provide evidence for clinical prevention and treatment of T2D-related bone diseases.

## Background

The prevalence of diabetes is rising rapidly. Recent studies estimate that about 463 million adults (20–79 years old) suffer from diabetes worldwide, of which about 90% are T2D [1]. T2D may cause various chronic complications, leading to high disability and high mortality [2]. Diabetes has destructive effects on the musculoskeletal system of patients, such as decreased bone formation and delayed bone healing [3, 4]. The fracture risk of T2D patients is also higher than that of normal people [5]. At present, it is getting more and more attention to explore the effect of diabetes on bone [6].

The increased risk of T2D fractures is associated with multiple factors. The BMD of T2D patients may be less than, equal to or even greater than the normal level, but the risk of fracture is increased [6–8]. Although BMD is a determinant of fracture risk [9], changes in bone structure and mineral composition caused by T2D may lead to the reduction of fracture resistance, thereby increasing the risk of fracture [10, 11]. In addition, various complications (such as cardiovascular disease, renal failure, peripheral neuropathy, and impaired vision, etc.) caused by T2D can also indirectly lead to an increase in the risk of fractures [12–14]. Bone has a multi-level structure, and comprehensive evaluation of the mechanical properties of bone can be achieved through multi-level evaluation methods, including compression test, microstructure analysis and finite element analysis [15]. It can help us better understand the mechanism of diabetes on bone to study the effect of diabetes on bone mechanical properties from different levels.

At present, there are a variety of drugs that can be used to treat T2D, it has not been fully demonstrated which drug treatment can effectively control diabetic bone disease [16–20]. Insulin is a commonly used medicine for diabetic patients, and different injection methods have different effects on blood glucose control. When oral hypoglycemic drugs fail and blood glucose control is poor, patients with T2D need to be treated with insulin injections [21]. Insulin mainly relies on exogenous infusion. There are two main ways to inject exogenous insulin, namely multiple daily insulin (MDI) and CSII. MDI is injected multiple times a day with a syringe, and CSII works by injecting insulin into a pump, which is then implanted into the body for continuous infusion [22, 23]. Studies have shown that both CSII and MDI can reduce the HbA1c level of T2D patients and maintain body weight unchanged or slightly increase. However, compared with MDI, CSII can achieve a lower HbA1c level with a lower total daily dose of insulin [24].

CSII treatment is not only superior to MDI treatment in blood glucose control, but also can improve the structure and mechanical properties of diabetic bone. In early T1D, CSII treatment maintained the normal structure and strength, while delayed CSII treatment only partially restored the structure and strength of femoral cortical and cancellous bones in mice [25]. For T2D, in a previous study of our group, 4-week and 8-week CSII treatments are found to improve the microstructure, mineral composition and macro-nano mechanical properties of the femur of T2D rats by affecting bone metabolism, bone formation and bone resorption, and 8-week CSII treatment is more effective than 4-week CSII treatment [26]. T2D patients have different fracture probabilities at different sites [7]. The effect of CSII treatment on femur was only considered in the previous study, but lumbar spine was not assessed. Lumbar spine is rich in cancellous bone. Cancellous bone is more metabolically active than cortical bone and more sensitive to drug stimulation [27]. In addition, T2D is closely related to vertebral fractures, intervertebral disc degeneration and severe chronic spinal pain [28]. Severe vertebral fractures in T2D patients are associated with higher mortality [29]. Therefore, based on the previous work [26]. the lumbar spines were selected to investigate the effects of 4-week and 8-week CSII treatments on the microstructures, mineral compositions and nanoscopic-mesoscopic-apparent-macroscopic mechanical properties of lumbar spines in T2D rats through nonlinear finite element analysis, microstructure analysis, nanoindentation test, compression test and Raman spectroscopy.

## Methods

### Animals, feeds and reagents

Seventy 6-week-old male SD rats with body weight of 200 ± 20 g were used for this study (Vital River Laboratory Animal Technology Co., Ltd., Beijing, China). High fat and high sugar Feed formula was 10% lard, 20% sucrose, 2% cholesterol, 1% cholate and 67% basic feed (Keao Xieli Feed Co., Ltd., Beijing, China). Main reagents and blood glucose measuring device including Streptozotocin (STZ, Solarbio science and technology co., Ltd., Beijing, China), insulin, ALZET osmotic pump (Model 2ML4, ALZET^®^ Osmotic Pumps, DURECT Corp., CA, USA), citrate buffer and blood glucose meter (Roche Diagnostics Co., Ltd., Shanghai, China). The rats were fed in an environment with natural lighting, indoor room temperature of 20℃–25℃ and relative humidity of 40%-70%. Animals can eat and drink freely during the experiment.

### Animal experiment

The animal experiment in this study was divided into two batches. The difference was that the treatment duration of CSII was 4 weeks and 8 weeks, respectively. They were referred to as 4-week and 8-week CSII treatments. Lumbar spine samples used in this study were from the same rats as the femur samples previously used in our laboratory [26].

**Fig. 1:**
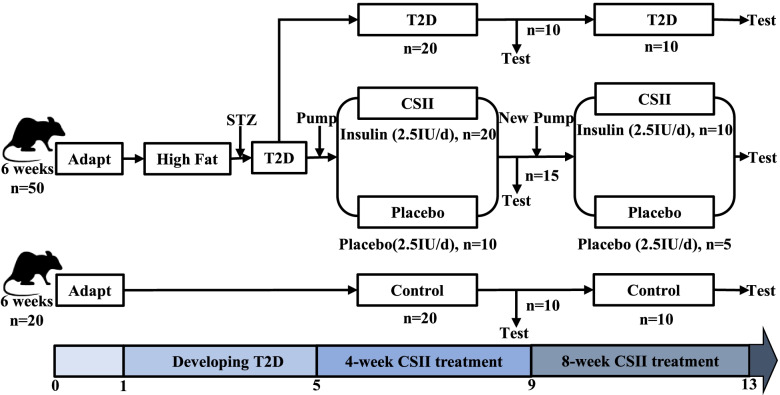
Process diagram of T2D rat modeling and insulin administration. Control: healthy control group; *T2D* type 2 diabetes; *CSII* continuous subcutaneous insulin infusion; *STZ* Streptozotocin; Placebo: citrate buffer

Process of T2D rat modeling and insulin administration was shown in Fig. 1. After adaptive feeding for one week, seventy 6-week-old male SD rats were randomly divided into Control (*n* = 20), T2D (*n* = 20), CSII (*n* = 20) and Placebo (*n* = 10) groups. Control rats were fed with normal diet, while the T2D, CSII and Placebo rats were fed with high fat and high sugar diet during the entire experimental period. After feeding for 4 weeks and fasting for 12-16 h (without water), the rats were intraperitoneally injected with 40 mg/kg STZ solution [30]. Non-fasting blood glucose concentration ≥ 16.7 mmol/L for at least three consecutive days was considered as T2D. AlZET osmotic pump was implanted subcutaneously in the back of CSII rats to inject insulin at 2.5 IU/ day. For Placebo rats, citrate buffer at equal dose was injected using an ALZET Osmotic Pump. After 4 weeks, half of the rats randomly selected from groups of Control, T2D, CSII and Placebo were sacrificed. The 2-3^th^ lumbar spine (L2-3) was isolated and stored at -20℃. The remaining rats were fed for another 4 weeks according to the above method, and then sacrificed to obtain L2-3 samples. Due to the structural characteristics of rat lumbar spine, L3 has more cancellous bone than L2. so it is easier to observe the influence of T2D and CSII on the microstructure of lumbar cancellous bone by micro-CT scanning of L3. CT images of the L3 were used to perform nonlinear finite element analysis to obtain mesoscopic and apparent mechanical properties of the lumbar spine. The material properties assigned to the L3 model need to be obtained by nanoindentation test, which is used to obtain the nanoscopic mechanical properties of the lumbar spine. To investigate the effects of T2D and CSII on the mineral composition of the lumbar spine, Raman spectroscopy was performed on samples from the nanoindentation test. In order to investigate the effects of T2D and CSII on the macroscopic mechanical properties of lumbar spine, L2 were selected for compression test.

### Micro-CT scanning

**Fig. 2:**
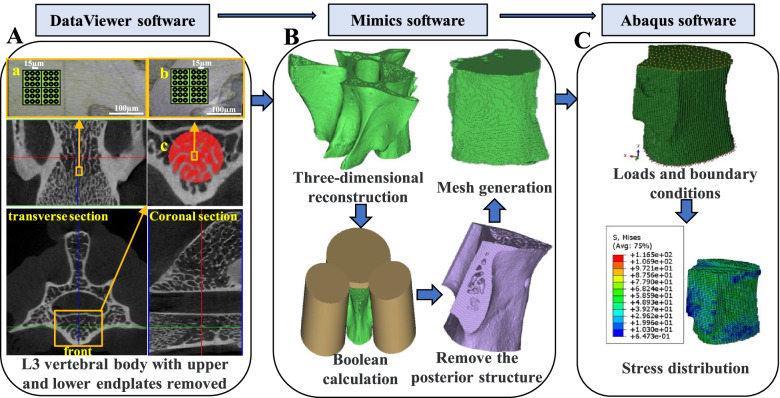
Flow chart of nonlinear finite element analysis of L3 in rat. **A** L3 vertebral body with upper and lower endplates removed. **a** Region of Nanoindentation test of cancellous bone in transverse direction (The indenter is perpendicular to the axial direction of longitudinal trabecula). **b** Region of Nanoindentation test of cancellous bone in longitudinal direction (The indenter is along the axial direction of longitudinal trabecula). **c** Region of interest of cancellous bone. **B** Three-dimensional reconstruction. **C** The nonlinear finite element analysis

The L3 of each rat was scanned by micro-CT system (Skyscan 1076, Skyscan, Belgium) with the scanning parameters of 70 kV, 141μA, and layer thickness of 18 μm. The voxel size was 18 × 18 × 18μm^3^. The TIF images of L3 of each rat were obtained after scanning. NRecon software was used to reconstruct the images, and the BMP images of L3 of rats were obtained. DataViewer software was used to remove the upper and lower endplates of L3 of each rat. The microstructures of L3 without upper and lower endplates were analyzed by CTAn software. For L3 of each rat, the largest cylinder-shaped cancellous bone region was selected as the region of interest. The diameter of the cylinder was equal to the diameter of the largest cancellous bone that a circular tool can take in the smallest cross-section of the vertebral body (region c in Fig. 2A). The cylinder did not exceed the vertebral area and contained as much cancellous bone as possible. Subsequently, the microstructure analysis of the selected region of interest was carried out. The microstructure parameters of the cancellous bone in the region of interest were obtained, including BV/TV, Tb.Th, BMD, trabecular number (Tb.N), trabecular separation (Tb.Sp) and structural model index (SMI).

### Nanoindentation test

The L3 of rats in each group were used for nano indentation test. After removing the upper and lower endplates, longitudinal cancellous bone sample with a thickness of 2 mm was cut from the upper surface of the vertebral body along the median coronal section for nanoindentation test in longitudinal directions (Fig. 2A). A 2 mm-thick transverse cancellous bone sample was cut from the front of the vertebral body along the median transverse section for nanoindentation test in the transverse direction (Fig. 2A). After all samples were dehydrated in gradient alcohol, they were embedded in epoxy resin, and the embedded samples were polished step by step with metallographic silicon carbide sandpaper to obtain the smooth surface required for nanoindentation test. The sample was placed on the horizontal tray under the microscope and the indenter, and adjust the position of the horizontal tray and the height of the sample until the clearest sample image was detected under the microscope. In this study, Nano Indenter G200 (Agilent Technologies, Inc., USA) was used for testing. The indentation depth was 580 nm, and the Poisson's ratio of bone was 0.3 [31]. The waiting time for the start of the experiment was 1.5 h. The data was corrected by establishing the thermal drift of the machine and the sample. Four indentation regions in each sample were selected, and each region was made 9 indentations with interval of 15 µm. The indentation areas were selected in the middle of the transverse and longitudinal cancellous bone samples, and all samples were loaded in the same direction (areas a and b in Fig. 2A). The indentation modulus (*E*_*b*_) and hardness (*H*) of bone materials were measured by the method of Oliver and Pharr [32], and the relevant formula was as follows:

1 $${E}_{b}=\frac{(1-{v}_{b}^{2}){E}_{i}\sqrt{\pi }S}{2\beta \sqrt{A}{E}_{i}-\sqrt{\pi }S(1-{v}_{i}^{2})}$$

2 $$H=\frac{{P}_{max}}{A}$$

where ${v}_{b}=0.3$ is the Poisson's ratio of bone, *A* is the contact area, S is the contact stiffness, ${P}_{max}$ is the peak load. ${v}_{i}=0.07$ and ${E}_{i}=1140GPa$ are the Poisson's ratio and elastic modulus of the diamond indenter used in the test. $\beta =1.034$ is a constant of the diamond indenter used in the test [32]. In the process of nanoindentation test, the interference of external factors such as noise and vibration should be avoided as far as possible. All experiments were carried out at night, and the consistency of indentation position of test samples should be ensured as far as possible to avoid the scratches produced by surface grinding.

### Raman spectroscopy

Raman spectra were obtained by using the LabRAM HR Evolution High resolution Raman spectrometer (HORIBA Scientific, Edison, NJ, USA) from the samples used for nanoindentation test. The excitation wavelength was 532 nm, the objective lens was 50 × , and the scanning spectra ranged from 600 to 2000 cm^−1^. The wave peaks of five spectral bands were extracted, and the range of each peak was determined by referring to other investigations, which was consistent in all spectra [33, 34]. Finally, the ratio of each integral area was calculated. PO_4_^3−^ ν1/Amide I, PO_4_^3−^ ν1/CH_2_ wag and PO_4_^3−^ ν1/Amide III represented mineral-to-matrix ratio. Type B carbonate substitution was denoted by CO_3_^2−^ ν1/PO_4_^3−^ ν1. The 1/full width at half maxima (FWHM) of the PO_4_^3−^ν1 peak was used to describe crystallinity.

### Nonlinear finite element analysis

The images of L3 without the upper and lower endplates by DataViewer software were imported into Mimics 17.0 (Materialise, Inc., Belgium) for three-dimensional reconstruction. In Mimics 17.0 software, three cylinders were created and Boolean operation was performed respectively with the established L3 model to obtain the model without upper and lower endplates and posterior structures (Fig. 2B). The model was meshed into hexahedral elements based on the voxel, and the element size was five times the size of the voxel to reduce the requirements on computer performance and save computing time [35]. The meshed model was imported into Abaqus 6.14 (ABAQUS Inc., Providence, RI, USA) software. Elastic modulus was obtained based on nanoindentation test, and uniform elastic modulus was allocated to the model [36], and Poisson's ratio was 0.3 [37]. Nonlinear material properties were assigned with the four-parameter bilinear model to simulate the nonlinear mechanical behavior of solid bone material [38, 39]. A compressive displacement boundary condition of 30 steps with an equal length to produce 1.5% strain along the axial direction of the vertebral body was applied to the model. The apparent stress and strain of each step were calculated. In order to ensure that the model only moved in the axial direction, the degrees of freedom other than U3 on the upper surface were constrained, and the lower surface was completely fixed. Apparent stress was equal to the surface reaction force divided by the area of the constrained surface. Apparent elastic modulus was calculated from the slope of the initial linear part of the apparent stress–strain curve. The initial apparent yield point was determined by 0.2% offset method. The apparent stress, the percentage of trabecular bone yielded and the average von Mises stress of cancellous bone at the apparent yield point from the model were calculated. The nonlinear finite element analysis process of L3 in rat is shown in Fig. 2.

### Compression test

The L2 was selected for compression test. The muscles and tissues around the L2 were removed, and the upper and lower endplates and posterior structures were removed, so that the L2 became an approximate cylindrical vertebral body sample with two parallel planes and a height of 4-6 mm. The height of the L2 was measured with a vernier caliper. The upper and lower ends of the L2 and the reference object ($1\times 1\times 1$ cm^3^ cube) with known area was placed on the same plane to take photos. The images were imported into Photoshop software, and the surface area of the upper and lower ends of the L2 was calculated according to the relationship of pixels between the L2 and reference object. The compression test was carried out on Instron ElectroPuls E10000 (Instron, Norwood, MA, USA). The room temperature was 20°C–25°C. During the experiment, normal saline was sprayed on the surface of L2 to keep it moist. The maximum failure load of the L2 was determined by pre-test. The L2 was repeatedly loaded and unloaded for twenty times between 0 N and 30% of the maximum failure load to stabilize the data. Then the displacement boundary condition was applied to the L2 at a loading speed of 2 mm/min until it failed. The experimental data were recorded and the load-deformation curve was drawn. Maximum load, elastic limit load, maximum stress, elastic limit stress, elastic modulus and energy absorption capacity were calculated.

### Statistical analysis

Results in Control, T2D, CSII, and Placebo groups were expressed as median (interquartile range). SPSS 22.0 (IBM, Inc., USA) and OriginPro 2017 (OriginLab, Inc., USA) software were used for data analysis. Since the sample sizes were relatively small and not all data of the same parameter in different groups were normally distributed. Nonparametric test (Kruskal–wallis test) was used to analyze the independent variables to investigate the differences among groups. Kruskal–Wallis one-way ANOVA (k samples) was used for post hoc pairwise comparisons to analyze statistical significance of the different groups. Values of *P* < 0.05 were considered to be statistically significant.

## Results

### Effects of CSII on nanoscopic mechanical properties

**Fig. 3:**
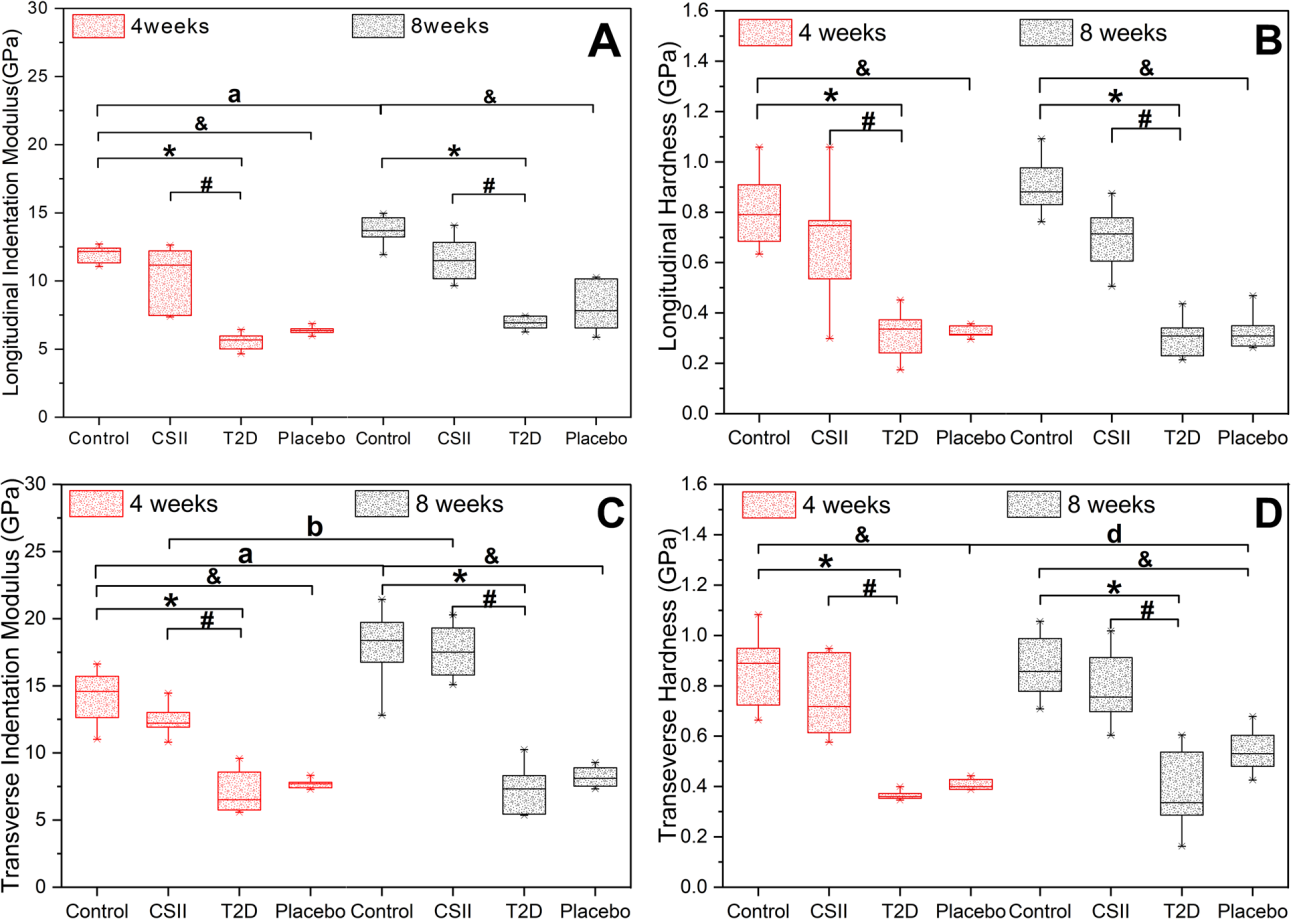
Nanoscopic mechanical properties of L3 cancellous bone in different groups of rats at 4-week and 8-week CSII treatments. **A** Longitudinal indentation modulus. **B** Longitudinal hardness. **C** Transverse indentation modulus. **D** Transverse hardness. ^*^ *P* < 0.05, T2D vs Control; ^#^ *P* < 0.05, CSII vs T2D; ^&^ *P* < 0.05, Placebo vs Control; ^a^ *P* < 0.05, Control (8 weeks) vs Control (4 weeks); ^b^ *P* < 0.05, CSII (8 weeks) vs CSII (4 weeks); ^d^ *P* < 0.05, Placebo (8 weeks) vs Placebo (4 weeks)

Results of nanoscopic mechanical properties of L3 cancellous bone in rats obtained by nanoindentation test were shown in Fig. 3. As shown in Fig. 3A-D, in 4-week and 8-week CSII treatments, the transverse and longitudinal indentation moduli and hardness of T2D group were significantly lower than those of Control and CSII groups (*P* < 0.05), and the transverse and longitudinal indentation moduli and hardness of Placebo group were both significantly lower than Control group (*P* < 0.05). As shown in Fig. 3A and Fig. 3C, in Control group, the transverse and longitudinal indentation moduli of 8-week CSII treatment were significantly higher than the corresponding parameters in 4-week CSII treatment (*P* < 0.05). In CSII group, the transverse indentation modulus of 8-week CSII treatment was significantly higher than that of 4-week CSII treatment (*P* < 0.05). As shown in Fig. 3D, the transverse hardness of 8-week CSII treatment in Placebo group was significantly higher than that of 4-week CSII treatment (*P* < 0.05).

### Effects of CSII on mesoscopic-apparent mechanical properties

**Fig. 4:**
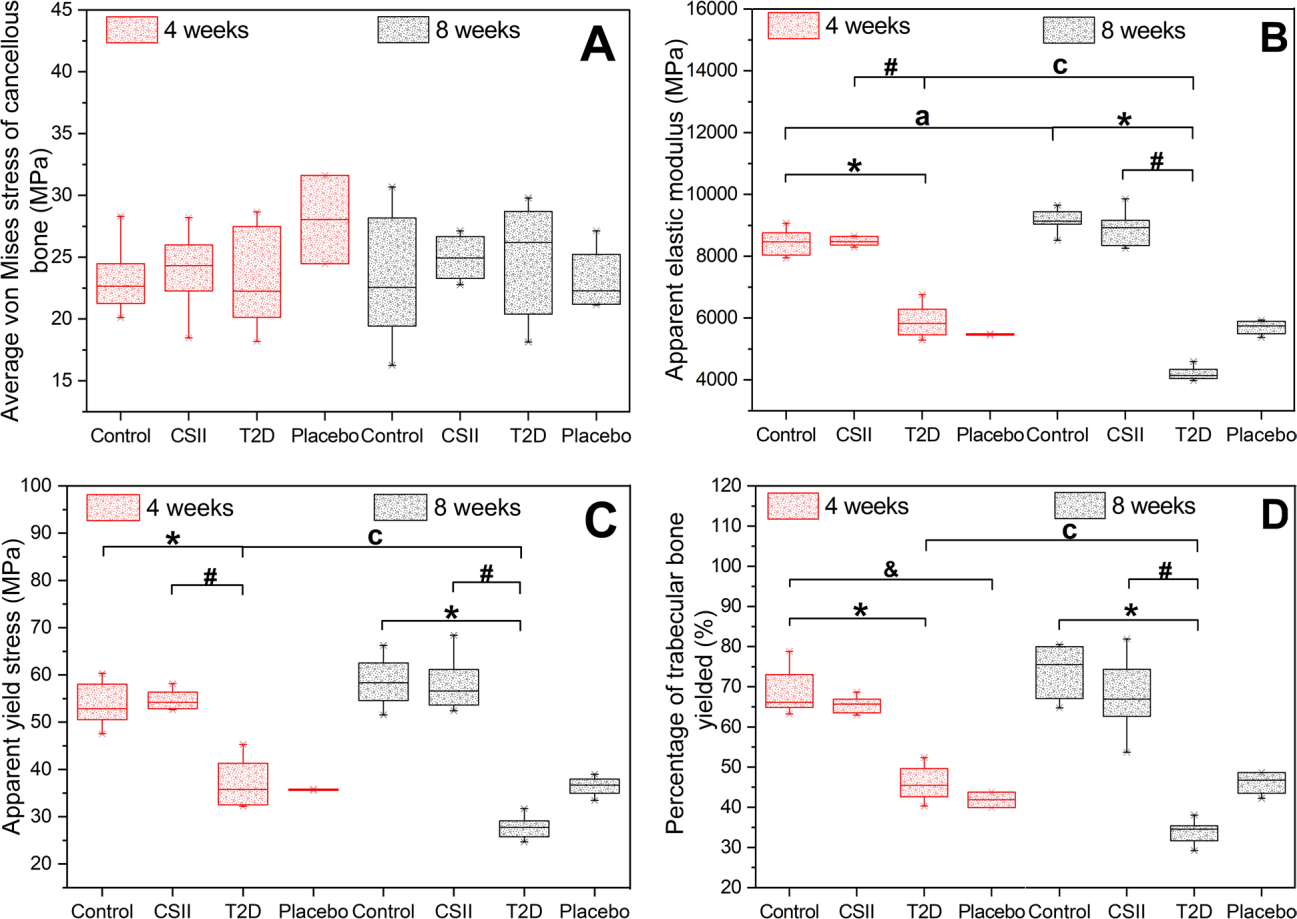
Mesoscopic-apparent mechanical properties of L3 in different groups of rats at 4-week and 8-week CSII treatments. **A** Average von Mises stress of cancellous bone. **B** Apparent elastic modulus. **C** Apparent yield stress. **D** Percentage of trabecular bone yielded. ^*^ *P* < 0.05, T2D vs Control; ^#^ *P* < 0.05, CSII vs T2D; ^&^ *P* < 0.05, Placebo vs Control; ^a^ *P* < 0.05, Control (8 weeks) vs Control (4 weeks); ^c^ *P* < 0.05, T2D (8 weeks) vs T2D (4 weeks)

Results of mesoscopic-apparent mechanical properties of L3 in different groups of rats obtained by nonlinear finite element analysis in 4-week and 8-week CSII treatments were shown in Fig. 4. As shown in Fig. 4A, there was no significant difference in the average von Mises stress of cancellous bone among groups (*P* > 0.05). As shown in Fig. 4B-C, in 4-week and 8-week CSII treatments, the apparent elastic modulus and apparent yield stress of T2D group were significantly less than those of Control and CSII groups (*P* < 0.05). In T2D group, the apparent elastic modulus and apparent yield stress in 8-week CSII treatment were significantly less than the 4-week CSII treatment. In Control group, the apparent elastic modulus in 8-week CSII treatment was significantly greater than that in 4-week CSII treatment (*P* < 0.05). It was observed in Fig. 4D that in 4-week CSII treatment, the percentage of trabecular bone yielded in T2D and Placebo groups was significantly less than in Control group (*P* < 0.05). In 8-week CSII treatment, the percentage of trabecular bone yielded in T2D group was significantly less than that in Control and CSII groups (*P* < 0.05). In T2D group, the percentage of trabecular bone yielded in the 8-week CSII treatment was significantly less than that in 4-week CSII treatment (*P* < 0.05).

### Effects of CSII on the microstructure parameters of cancellous bone

In 4-week and 8-week CSII treatments, the parameters of microstructure of L3 cancellous bone of rats in different groups obtained by micro-CT scanning were shown in Table 1. In 4-week and 8-week CSII treatments, there were no significant differences in all parameters between CSII and Control groups (*P* > 0.05). In 4-week CSII treatment, Tb.Th in T2D group was significantly lower than that in Control and Placebo groups (*P* < 0.05). In 8-week CSII treatment, BMD, BV/TV and Tb.Th in T2D group were significantly lower than those in Control group (*P* < 0.05).

**Table 1:** Microstructure parameters of L3 in different groups of rats at 4-week and 8-week of CSII treatments

	Treatment time	Control	CSII	T2D	Placebo
BMD (g cm^−3^)	4 weeks	0.20 (0.18–0.20)	0.18 (0.18–0.20)	0.18 (0.17–0.19)	0.21 (0.20–0.21)
	8 weeks	0.21 (0.19–0.23)	0.18 (0.17–0.21)	0.16 (0.15–0.19) ^*^	0.23 (0.20–0.25)
BV/TV (%)	4 weeks	27.88 (26.36–30.39)	26.99 (24.81–31.27)	26.86 (24.35–27.73)	32.88 (32.27–33.48)
	8 weeks	31.71 (28.33–35.89)	27.23 (25.71–31.79)	23.43 (22.07–26.90) ^*^	36.50 (30.49–39.01)
SMI	4 weeks	0.84 (0.81–1.17)	0.96 (0.76–1.11)	1.13 (1.04–1.25)	0.78 (0.78–0.78)
	8 weeks	0.77 (0.50–0.85)	0.84 (0.81–1.17)	1.20 (1.08–1.33)	0.38 (0.17–0.88)
Tb.Th (mm)	4 weeks	0.09 (0.09–0.09)	0.09 (0.09–0.09)	0.08 (0.08–0.09) ^*^	0.10 (0.10–0.10) ^@^
	8 weeks	0.10 (0.09–0.10)	0.09 (0.08–0.09)	0.08 (0.08–0.08) ^*^	0.10 (0.09–0.11)
Tb.N (mm^−1^)	4 weeks	3.27 (3.18–3.38)	3.10 (3.02–3.44)	3.13 (3.05–3.28)	3.20 (3.17–3.23)
	8 weeks	3.33 (3.32–3.45)	3.11 (2.90–3.38)	3.04 (2.79–3.15)	3.54 (3.26–3.56)
Tb.Sp (mm)	4 weeks	0.24 (0.22–0.25)	0.24 (0.23–0.25)	0.25 (0.24–0.26)	0.23 (0.22–0.23)
	8 weeks	0.23 (0.22–0.24)	0.24 (0.23–0.27)	0.25 (0.23–0.28)	0.22 (0.21–0.23)

Values were expressed as median (interquartile range). ^*^ *P* < 0.05, T2D vs Control; ^@^ *P* < 0.05, Placebo vs T2D

### Effects of CSII on macroscopic mechanical properties

**Fig. 5:**
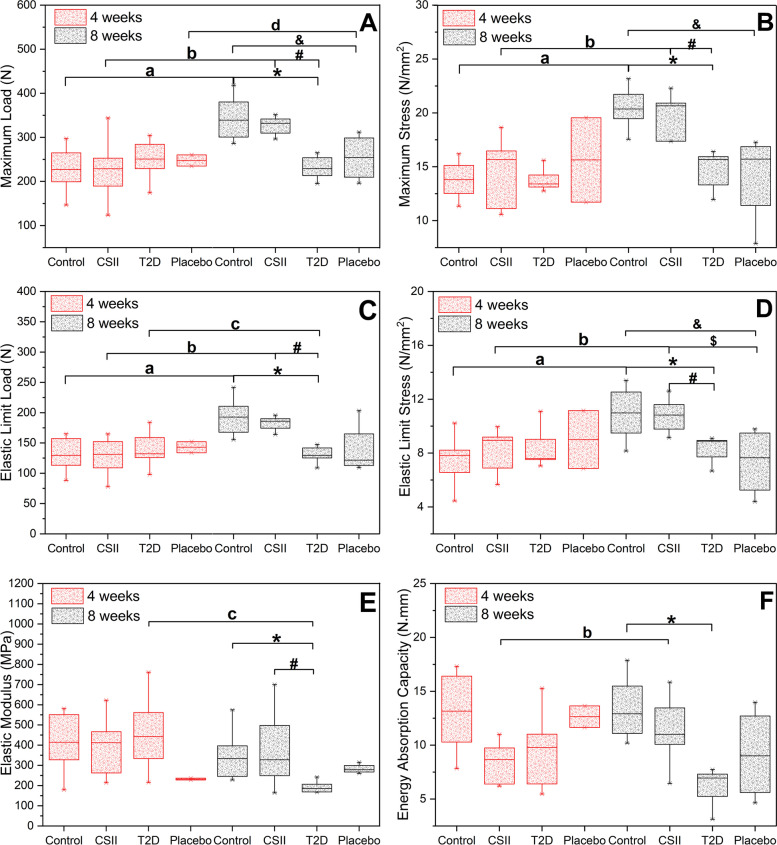
Macroscopic mechanical properties of L2 in different groups of rats at 4-week and 8-week CSII treatments. **A** Maximum load. **B** Maximum stress. **C** Elastic limit load. **D** Elastic limit stress. **E** Elastic modulus. **F** Energy absorption capacity. ^*^ *P* < 0.05, T2D vs Control; ^#^ *P* < 0.05, CSII vs T2D; ^&^ *P* < 0.05, Placebo vs Control; ^$^ *P* < 0.05, Placebo vs CSII, ^a^ *P* < 0.05, Control (8 weeks) vs Control (4 weeks); ^b^ *P* < 0.05, CSII (8 weeks) vs CSII (4 weeks); ^c^ *P* < 0.05, T2D (8 weeks) vs T2D (4 weeks); ^d^ *P* < 0.05, Placebo (8 weeks) vs Placebo (4 weeks)

Results of macroscopic mechanical properties of L2 in different groups of rats at 4-week and 8-week CSII treatments obtained by compression test were shown in Fig. 5. As shown in Fig. 5A-F, in 4-week CSII treatment, there were no significant differences in the maximum load, maximum stress, elastic limit load, elastic limit stress, elastic modulus and energy absorption capacity among all groups (*P* > 0.05). At 8-week CSII treatment, the above parameters in T2D group were significantly smaller than those in Control and CSII groups except for energy absorption capacity (*P* < 0.05), and the energy absorption capacity in T2D group was significantly smaller than that in Control group (*P* < 0.05). The maximum load, maximum stress and elastic limit stress of Placebo group were significantly smaller than those of Control group (*P* < 0.05), and the elastic limit stress of Placebo group was significantly lower than that of CSII group (*P* < 0.05). In Control and CSII groups, the maximum load, maximum stress, elastic limit load and elastic limit stress of 8-week CSII treatment were significantly higher than the corresponding parameters of 4-week CSII treatment (*P* < 0.05). In 8-week CSII treatment, the energy absorption capacity of CSII group, the maximum load of Placebo group, the elastic limit load and elastic modulus of T2D group were significantly different from those in 4-week CSII treatment (*P* < 0.05).

### Effects of CSII on bone mineral compositions

**Fig. 6:**
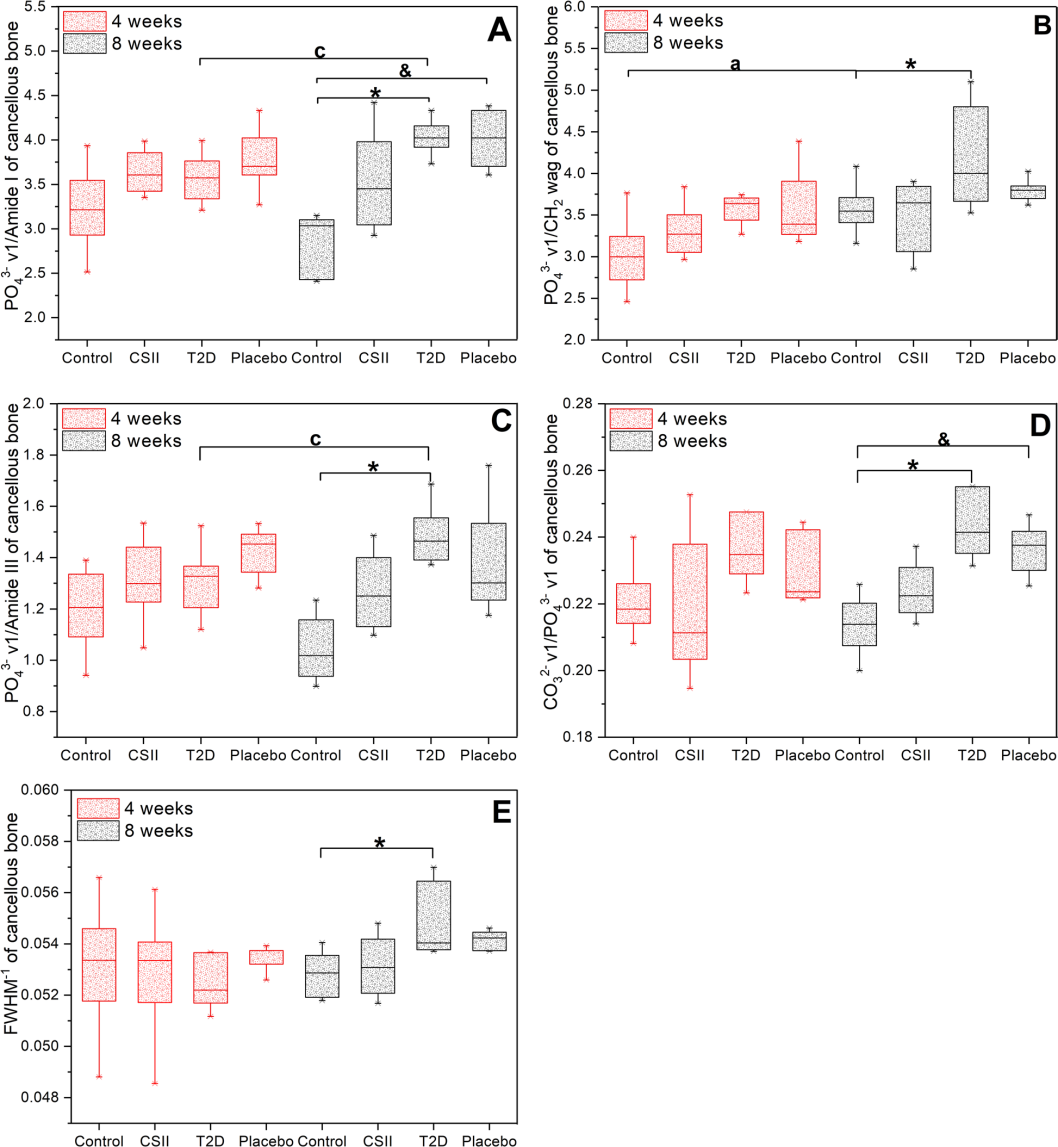
Mineral compositions of L2 cancellous bone in different groups of rats at 4-week and 8-week CSII treatments. **A** PO_4_^3 −^ ν1/Amide I. **B** PO_4_^3 −^ ν1/CH_2_ wag. **C** PO_4_^3 −^ ν1/Amide III. **D** CO_3_^2 −^ ν1/ PO_4_^3 −^ ν1. **E** FWHM^-1^. ^*^ *P* < 0.05, T2D vs Control; ^&^ *P* < 0.05, Placebo vs Control; ^a^ *P* < 0.05, Control (8 weeks) vs Control (4 weeks); ^c^ *P* < 0.05, T2D (8 weeks) vs T2D (4 weeks)

**Fig. 7:**
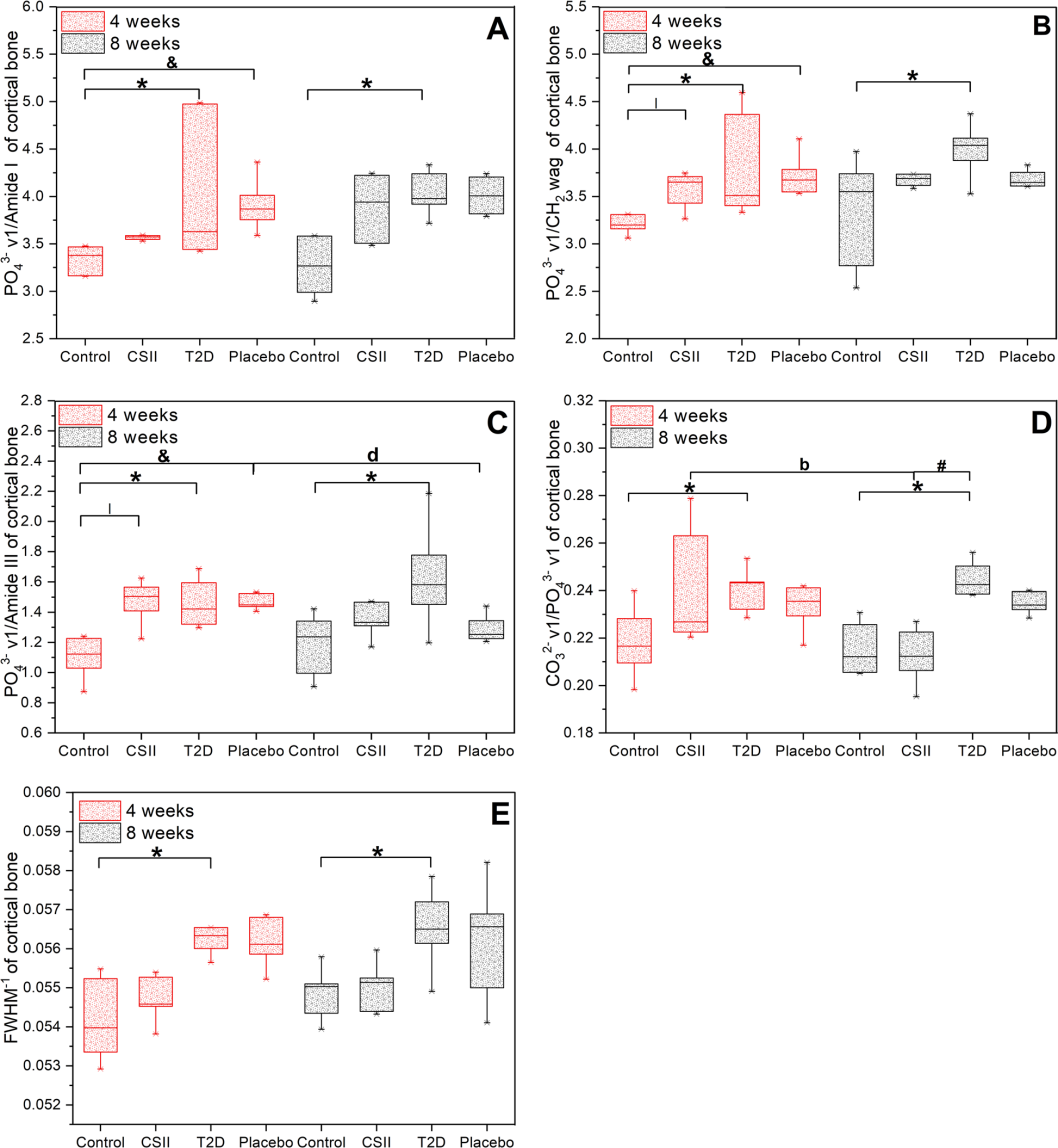
Mineral compositions of L2 cortical bone in different groups of rats at 4-week and 8-week CSII treatment. **A** PO_4_^3 −^ ν1/Amide I. **B** PO_4_^3 −^ ν1/CH_2_ wag. **C** PO_4_^3 −^ ν1/Amide III. **D** CO_3_^2 −^ ν1/ PO_4_^3 −^ ν1. **E** FWHM^-1^. ^*^ *P* < 0.05, T2D vs Control; ^#^ *P* < 0.05, CSII vs T2D; ^&^ *P* < 0.05, Placebo vs Control; *P* < 0.05, Control vs CSII, ^b^ *P* < 0.05, CSII (8 weeks) vs CSII (4 weeks); ^d^ *P* < 0.05, Placebo (8 weeks) vs Placebo (4 weeks)

Mineral compositions of L3 cancellous and cortical bone from different groups of rats obtained by Raman spectroscopy were shown in Fig. 6 and Fig. 7. In cancellous bone, there were no significant differences in parameters among all groups in 4-week CSII treatment (*P* > 0.05). At 8-week CSII treatment, the mineral-to-matrix ratio (PO_4_^3−^ ν1/Amide I, PO_4_^3−^ ν1/CH_2_ wag and PO_4_^3−^ ν1/Amide III), crystallinity (FWHM^−1^) and type B carbonate substitution (CO_3_^2−^ ν1/PO_4_^3−^ ν1) in T2D group were significantly higher than those in Control group (*P* < 0.05). PO_4_^3−^ ν1/Amide I and type B carbonate substitution of Placebo group were significantly higher than those of Control group (*P* < 0.05). In Control group, the PO_4_^3−^ ν1/CH_2_ wag of 4-week CSII treatment was significantly lower than that of 8-week CSII treatment (*P* < 0.05). In T2D group, PO_4_^3−^ ν1/Amide I and PO_4_^3−^ ν1/Amide III in 4-week CSII treatment were significantly lower than those in 8-week CSII treatment (*P* < 0.05). In cortical bone, at 4-week CSII treatment, the mineral-to-matrix ratio in both T2D and Placebo groups was significantly higher than Control group, and type B carbonate substitution and crystallinity of T2D group were significantly higher than Control group, PO_4_^3−^ ν1/CH_2_ wag and PO_4_^3−^ ν1/Amide III in CSII group were significantly higher than those of Control group (*P* < 0.05). In 8-week CSII treatment, the mineral-to-matrix ratio, type B carbonate substitution and crystallinity of T2D group were significantly higher than those of Control group (*P* < 0.05). The type B carbonate substitution in T2D group was significantly higher than that in CSII group (*P* < 0.05). In Placebo group, PO_4_^3−^ ν1/Amide III in 4-week CSII treatment was significantly higher than that of 8-week CSII treatment (*P* < 0.05). In CSII group, the type B carbonate substitution at 4-week CSII treatment was significantly higher than that in 8-week CSII treatment (*P* < 0.05).

## Discussion

CSII treatment could improve the structure and mechanical properties of the femur of T2D rats by affecting bone remodeling [26], while the effect on the lumbar spine of T2D rats remains unclear. The effects of 4-week and 8-week of CSII treatments on the microstructure, bone mineral composition and nanoscopic-mesoscopic-apparent-macroscopic mechanical properties of the lumbar spine of T2D rats were investigated in this study. It was observed that T2D had adverse effects on the microstructure, nanoscopic-mesoscopic-apparent-macroscopic mechanical properties and bone mineral composition of the lumbar spine. CSII treatment could significantly improve the damage of nanoscopic, apparent, partial mesoscopic (percentage of trabecular bone yielded in Fig. 4) and some macroscopic mechanical properties (maximum load, elastic limit load, maximum stress, elastic limit stress and elastic modulus in Fig. 5) (*P* < 0.05). It also improved the microstructure and bone mineral composition of lumbar spine, but the effect was not significant (*P* > 0.05). The adverse effects of T2D and improvements of CSII treatment on cancellous bone mineral composition (Fig. 6), BMD, BV/TV (Table 1) and macro-mechanical properties (Fig. 5) of lumbar spine all appeared after 8 weeks. In addition, the effects of T2D and CSII treatment on bone mineral compositions of lumbar spine cancellous bone in T2D rats were later than those of cortical bone.

Micro-CT analysis showed that BMD, Tb.Th and BV/TV were significantly decreased in lumbar spine of T2D rats (Table 1). T2D leads to the decrease of Tb.Th and BV/TV in lumbar spine cancellous bone, which was consistent with the previous studies [40, 41]. However, the changes in BMD caused by T2D were still controversial. Studies on rats and patients of T2D have shown that BMD may be normal, decreased or increased [6–8, 26, 42, 43], but both rats and patients of T2D have significantly higher fracture risk than the normal group, because bone strength and fracture risk were determined by BMD and bone structure [9]. Differences in BMD might be caused by the complex pathogenesis of T2D [44], which is related to drugs, duration of T2D and multiple chronic complications [2]. Our study supported that T2D led to decreased BMD, Tb.Th and BV/TV, and CSII treatment could improve BMD and bone microstructure, but it would take longer treatment time to recover the changes in bone microstructure caused by T2D.

Furthermore, we found that Tb.Th in T2D group was significantly lower than that Placebo groups after 4 weeks of CSII treatment (Table 1). It may be related to the injected citrate in the Placebo group. In fact, previous study has shown that high concentrations of citrate exist in bone humans and all osteo-vertebrates [45]. However, citrate content of bone is reduced in osteoporotic rats [46]. The addition of citrate to non-modified cement implanted in the defect of proximal tibial in rat appears to promote bone remodeling and bone formation at the early stage of bone healing [47]. Therefore, in the early stage of T2D, lumbar vertebral bone formation may be increased in Placebo groups due to citrate input, which partially restores the structure of cancellous bone.

Ultimate stress, ultimate strain, elastic modulus and energy absorption capacity were important factors for evaluating bone strength [48]. Increased crystallinity, mineralization defects (hypo- or hypermineralization) and collagen deformation all led to changes in the above parameters [49–52]. T2D increased the crystal size, that is, the crystallinity increased [53, 54]. A previous study in our group has also shown that T2D led to increased femur crystallinity and mineral-to-matrix ratio in rats [26]. In this study, we found that bone mineral composition (crystallinity, type B carbonate substitution and mineral-to-matrix ratio in Fig. 6 and Fig. 7) of lumbar cortical and cancellous bones of rats in T2D group significantly increased after 8 weeks compared with Control group (*P* < 0.05). BMD, Tb.Th, BV/TV (Table 1) and macroscopic mechanical properties (maximum load, elastic limit load, maximum stress, elastic limit stress, elastic modulus and energy absorption capacity in Fig. 5) were significantly decreased (*P* < 0.05), but there were no significant differences in CSII group (*P* > 0.05). These results suggested that T2D reduced bone strength by affecting BMD, structure, and bone mineral composition, and CSII therapy could ameliorate the negative effects of T2D.

CSII treatment improved the microstructure, mineral composition, and nanoscopic-macroscopic mechanical properties of the lumbar spine in T2D rats. This was consistent with our previous observations in femurs [26]. Unlike femur, the adverse effects of T2D on lumbar BMD, BV/TV, macro-mechanical properties, and mineral composition of cancellous bone, as well as the treatment effect of CSII, only showed up after 8 weeks. In addition, bone mineral composition of lumbar cancellous bone was affected by T2D and CSII later than cortical bone. On the one hand, this may be related to differences in the function and structure of bone tissues in different anatomical regions of the same species. In a previous study, micro-CT was used to assess BMD and microstructure of the areas of interest of cancellous bone in tibia, femur, lumbar spine and mandible of rats with diabetes after 4, 8, and 12 weeks, and it was found that areas with denser cancellous bone were less influenced by diabetes and the time was later [41]. In addition, Goodyear et al. compared the mineral composition of cortical and cancellous bone from standard laboratory mice and found that type B carbonate substitution and mineral-to-matrix ratio in cortical bone was significantly larger than cancellous bone [55]. On the other hand, our study has further confirmed that not all bone sites in rats experience bone loss at the same rate [56]. For example, significant bone loss was observed in the lumbar spine cancellous bone one month later than in the femoral neck in ovariectomized rats [57]. However, our study suggested that T2D and CSII treatment affected the lumbar cancellous bone structure, bone mineral composition and macroscopic mechanical properties in a time-dependent manner, that is, the bone mineral composition of the lumbar spine cortical bone was first affected, the cancellous bone structure and bone mineral composition were the second, and finally, the macroscopic mechanical properties was affected.

The apparent and meso-mechanical properties of the bone structure can be obtained by finite element analysis based on micro-CT images. The apparent mechanical properties of bone structure include apparent elastic modulus, apparent yield strain and apparent yield stress. The mechanical properties at the tissue level can be described by the average von Mises stress of cancellous bone and the percentage of trabecular bone yielded [58]. The mechanical properties of bone structure are determined by the mechanical properties and microstructure of bone materials, which can be obtained by nanoindentation test and micro-CT scanning. In this study, it was shown that T2D significantly reduced the microstructure (BV/TV and Tb.Th in Table 1), the nanoscopic mechanical properties (indentation modulus and hardness in Fig. 3) and the apparent mechanical properties (apparent elastic modulus and apparent yield stress in Fig. 4) of the bone materials (*P* < 0.05). Similar results have been found in related studies [59]. In addition, our study also found that T2D resulted in a significant decrease in mesoscopic mechanical properties (percentage of trabecular bone yielded), while CSII treatment significantly improved the adverse effects of nanoscopic and apparent mechanical properties that led by T2D (*P* < 0.05), and the improvement of mesoscopic mechanical properties was only significant after 8-week CSII treatment. It can also improve the microstructure of bone. In conclusion, CSII treatment can improve the mechanical properties of bone materials and cancellous bone structure damage caused by T2D, and thus significantly improve the mesoscopic-apparent mechanical properties.

There are some limitations in this study. Firstly, the effects of CSII treatment on the microstructure, bone mineral composition and multiscale mechanical properties of diabetic lumbar vertebrae were investigated in male rats. There was strong evidence that hyperglycemia causes the accumulation of advanced glycation endproducts (AGEs) to promote the activation of tumor necrosis factor α and osteoclast differentiation factor and improve osteoclast activity [60]. The detection of AGEs in bone tissue should be included in future experimental protocols to study the influence of AGEs content on bone structure and mechanical properties. Secondly, CSII treatment was observed to improve the microstructure and bone mineral composition of lumbar spine cancellous bone in rat after 8 weeks in this study, but not to a significant level. These results suggested that it was necessary to establish a longer time of CSII treatment to better understand the damage of T2D to bone and the repair and improvement effect of CSII treatment on bone. Finally, since estrogen deficiency led to massive cancellous bone loss in rats [61], further studies on female T2D animals need to be carried out to determine the effects of T2D and CSII treatment on bones of different genders.

## Conclusions

In summary, T2D rats showed significantly decreased lumbar BMD, Tb.Th, BV/TV, mechanical properties of nanoscopic (indentation modulus and hardness), microscopic (percentage of trabecular bone yielded), apparent (apparent elastic modulus and apparent yield stress) and macroscopic (maximum load, elastic limit load, maximum stress, elastic limit stress, elastic modulus and energy absorption capacity) (*P* < 0.05). And bone mineral composition (mineral-to-matrix ratio, type B carbonate substitution and crystallinity) of cortical and cancellous bones were significantly increased (*P* < 0.05). CSII treatment significantly improved the nanoscopic-mesoscopic-apparent-macroscopic mechanical parameters. BMD, bone microstructure and bone mineral composition can also be improved, but it would take longer treatment time to restore the normal level. The adverse effects of T2D and improvements of CSII treatment on bone mineral composition, BMD, BV/TV and macro-mechanical properties of lumbar spine appeared after 8 weeks. In addition, the effects of T2D and CSII treatment on bone mineral composition of lumbar spine cancellous bone in T2D rats were later than those of cortical bone. Our study can provide evidence for clinical prevention and treatment of T2D-related bone diseases.

## Data Availability

The datasets used during the present study are available from the corresponding author on reasonable request.

## Abbreviations

CSII: Continuous subcutaneous insulin infusion
T2D: Type 2 diabetes
SD: Sprague-Dawley
micro-CT: Micro-computed tomography
Tb Th: Trabecular thickness
BMD: Bone mineral density
BV/TV: Bone volume fraction
MDI: Multiple daily insulin
STZ: Streptozotocin
L2-3: The 2-3^th^ lumbar spine
Tb N: Trabecular number
Tb Sp: Trabecular separation
SMI: Structural model index
AGEs: Advanced glycation end products
